# Incidence and risk factors for cholelithiasis after bariatric surgery: a systematic review and meta-analysis

**DOI:** 10.1186/s12944-023-01774-7

**Published:** 2023-01-14

**Authors:** Yu Dai, Bujiangcun Luo, Weizheng Li

**Affiliations:** 1 Department of General Surgery, Third Xiangya Hospital, Central South University, No.138, Tongzipo Road, Yuelu District, Changsha City, Hunan Province China; 2 Xiangya School of Medicine, Central South University, Changsha City, Hunan Province China

**Keywords:** Bariatric surgery, Cholelithiasis, Risk factors, Meta-analysis

## Abstract

**Background:**

Obesity has been identified as an independent risk factor for cholelithiasis. As a treatment for obesity, bariatric surgery may increase the incidence of cholelithiasis. The risk factors for cholelithiasis after bariatric surgery remain uncertain. The purpose of this study was to explore the risk factors for postoperative cholelithiasis after weight-loss surgery and propose suggestions for clinical decision making.

**Methods:**

Four databases, PubMed, EMBASE, Web of Science and Cochrane, were systematically searched for all reports about cholelithiasis after bariatric surgery, and literature screening was performed following prespecified inclusion criteria. The included studies were all evaluated for quality according to the NOS scale. Data extraction was followed by analysis using Reviewer Manager 5.4 and StataSE 15.

**Results:**

A total of 19 articles were included in this meta-analysis, and all studies were of high quality. A total of 20,553 patients were included in this study. Sex [OR = 0.62, 95% CI (0.55, 0.71), *P* < 0.00001] and race [OR = 1.62, 95% CI (1.19, 2.19), *P* = 0.002] were risk factors for cholelithiasis after bariatric surgery. Surgical procedure, preoperative BMI, weight-loss ratio, smoking, hypertension, diabetes mellitus, and dyslipidemia were neither protective nor risk factors for cholelithiasis after bariatric surgery.

**Conclusion:**

Caucasian race and female sex are risk factors for developing cholelithiasis after bariatric surgery; surgical procedure, BMI, weight loss ratio, hypertension, diabetes mellitus, dyslipidemia, and smoking are not risk factors for cholelithiasis after bariatric surgery.

**Supplementary Information:**

The online version contains supplementary material available at 10.1186/s12944-023-01774-7.

## Introduction

Obesity is a significant health problem in the world today and is responsible for a large portion of health care expenditures in many Western countries [1]. Bariatric surgery is an important treatment for severe obesity, and a Food and Drug Administration (FDA)-approved obesity management guideline proposed that bariatric surgery be recommended for patients with BMI > 40 kg / m^2^ or BMI > 35 kg / m^2^ concurrent with severe obesity complications [2]. Bariatric surgery is recommended for obesity that cannot be controlled with diet and medications, especially in patients with type 2 diabetes, in whom Roux­en­Y gastric bypass (RYGB) is most effective [3]. Four surgical procedures are currently prevalent: the most frequently used and the gold standard procedure, the RYGB procedure; the more frequently used procedure in recent years, sleep gastrectomy (SG); adjustable gastric band, which has been indicated by multiple studies to be associated with a significantly higher complication rate than other surgical procedures [4] and thus has been gradually less frequently used; and the typical procedure for malabsorption, biliopancreatic diversion with duodenal switch (BPD–DS).

Obesity as well as its complications, such as insulin resistance and dyslipidemia, have been identified as independent risk factors for cholelithiasis [5]. Bariatric surgery has been shown to be effective for treating both obesity and its complications; however, bariatric surgery does not reduce the incidence of cholelithiasis. In contrast, many studies have found that bariatric surgery may increase the incidence of cholelithiasis [6]. Cholelithiasis is a severe complication requiring close attention after bariatric surgery, with 10% of patients undergoing RYGB or SG having to undergo cholecystectomy postoperatively due to severe cholelithiasis [7].

Cholelithiasis mainly results from the following four causes: cholesterol supersaturation in bile caused by excessive hepatic cholesterol secretion due to genetic factors; systolic dysfunction of the gallbladder wall; intestinal dysfunction with excessive absorption of cholesterol or cholesterol supersaturation aroused by disturbance in the hepatic circulation of bile [8]; and accelerated growth of cholesterol crystals and solid cholesterol crystals. The underlying mechanism is as follows: the liver secretes cholesterol into the bile, and the excess fraction is carried by lecithin cholesterol vesicles, within which cholesterol is high, has affinity and easily aggregates. These vesicles, when aggregated, eventually become the nuclei initiating the most aggregation of the stones [9]. Granulocytes are triggered after the formation of cholesterol crystals, which expel the DNA out of the cell and encapsulate cholesterol crystals, and then individual crystals aggregate to form larger stones [10]. Therefore, supersaturation of cholesterol is a necessary prerequisite for gallstones [5].

There are two possible reasons for the increasing incidence of cholelithiasis after bariatric surgery. One is that rapid weight loss causes fat mobilization and then a rise in serum cholesterol and triglyceride levels. On the other hand, intestinal dysfunction due to bariatric surgery with decreased cholecystokinin levels could cause gallbladder contractile dysfunction [11].

Epidemiological studies on cholelithiasis after bariatric surgery have been ongoing, and there have been previous meta-analyses based on randomized controlled trials (RCTs) investigating the preventive effect of ursodeoxycholic acid (UCDA) on cholelithiasis after bariatric surgery [12]. The aim of this article is to explore the full spectrum of accessible risk factors for concurrent cholelithiasis after bariatric surgery, with a view to clarify the association of relevant exposures with the incidence of postoperative cholelithiasis and to make recommendations for clinical decision making.

## Methods

### Protocols and registration

This meta-analysis is based on the Preferred Reporting Items for Systematic Reviews and Meta-Analyses (PRISMA) [13]. This study has been registered in the Prospective Register of Systematic Reviews (PROSPERO), and the protocol number is CRD42022332008.

### Information sources and search strategy

In April 2022, we systematically searched four databases, PubMed, Embase, Web of Science, and Cochrane, and obtained all original studies related to bariatric surgery, cholelithiasis, and risk factors. The literature search adopted the strategy of subject word search and free word search. The relevant keywords were as follows: bariatric surgery, cholelithiasis, risk factors. The basic logic of the search was (bariatric surgery) AND (cholelithiasis) AND (risk factors). Detailed search strategies can be found in Additional file 1. In addition, we performed manual retrieval for literature not included in online databases that was relevant to our study.

### Eligibility criteria

Studies included in this meta-analysis included all original studies for which the full text was available, whether published or unpublished. According to the PICOS principle, studies that met the following inclusion criteria were included: (I) patients undergoing bariatric surgery; (II) no preoperative symptoms of cholelithiasis; (III) no special interventions, especially currently recognized preventive ursodeoxycholic acid therapy; (IV) the articles compare the characteristics of patients with postoperative cholelithiasis and patients without postoperative cholelithiasis; (V) the articles provide OR values and 95% confidence intervals, or the number of different outcomes in each group is listed to facilitate the subsequent calculation of OR values and 95% confidence intervals; and (VI) cohort studies and case–control studies. Articles that did not meet the above criteria were excluded.

### Study selection

After retrieving all the literature, literature screening was performed using the following steps: first, duplicate literature was removed, then literature reviews, systematic reviews and meta-analyses were removed, and then animal experiments, guidelines, letters, reviews, and conference proceedings were screened out. Then, obviously irrelevant articles, preclinical studies, autopsy and case reports, cross-sectional studies, and clinical trials were removed by reading the article titles and abstracts. The full text of the remaining articles was obtained, and the articles for which the full text could not be obtained were removed through all accessible approaches. The full text was read to remove articles that did not meet the inclusion criteria described above. The literature screening was conducted independently by two authors, and when disagreements arose, a consensus was reached by consulting a third author to resolve the disagreement.

### Data extraction

After obtaining all the articles that met the preestablished inclusion criteria through literature screening, the two authors independently read all the articles and extracted relevant information of the patients with and without postoperative cholelithiasis, including author, country, publication year, study type, sex, preoperative body mass index (BMI), preoperative weight, surgery procedure, follow-up time, and age. The risk factors analyzed in this study included sex, race, preoperative BMI, surgical procedure, weight loss, hypertension, diabetes, dyslipidemia, and smoking. Therefore, the OR value and 95% confidence interval of the corresponding risk factors need to be extracted. For studies that did not provide OR values ​​and 95% confidence intervals, the corresponding data were extracted according to the type of study. For cohort studies, the specific number of postoperative complicated and uncomplicated cholelithiasis in the exposure group/nonexposure group were extracted. For case–control studies, the specific number of postoperative complicated and uncomplicated cholelithiasis cases in the case group/control group was extracted beforehand so that authors could calculate the OR value and 95% confidence interval in the study. All data extraction was performed independently by two authors (DY and LBJC), and when disagreements were encountered, the third author (LWZ) joined the discussion to reach a consensus.

### Quality assessment of studies

All studies included in this meta-analysis used the Newcastle Ottawa Scale (NOS scale) [14] to evaluate the quality of the literature. Literature with a score of 6 or more was defined as high-quality literature [15]. All authors reached consensus on the quality assessment of the literature.

### Statistical analysis

All data for this meta-analysis were analyzed in Reviewer Manager 5.4 and StataSE 15. We combined the OR and 95% confidence interval to explore whether exposure was a risk factor. Exposure was considered a risk factor when the OR was greater than 1 and the confidence interval did not include 1; otherwise, it was considered a protective factor. In addition, using the Q test and I^2^ test to evaluate the heterogeneity, I^2^ < 50% and *P* > 0.1 considered the heterogeneity acceptable. To be more scientific, sensitivity analysis was performed to evaluate the stability of the combined results and supplemented the publication bias analysis to check whether the article had publication bias. The symmetry of funnel diagram can directly reflect the publication bias, which can be found in additional file 3 and additional file 5. In addition, we used the Begg test and Egger’s test, and it was considered that there was no publication bias if *p* < 0.05.

## Results

### Search results

**Fig. 1:**
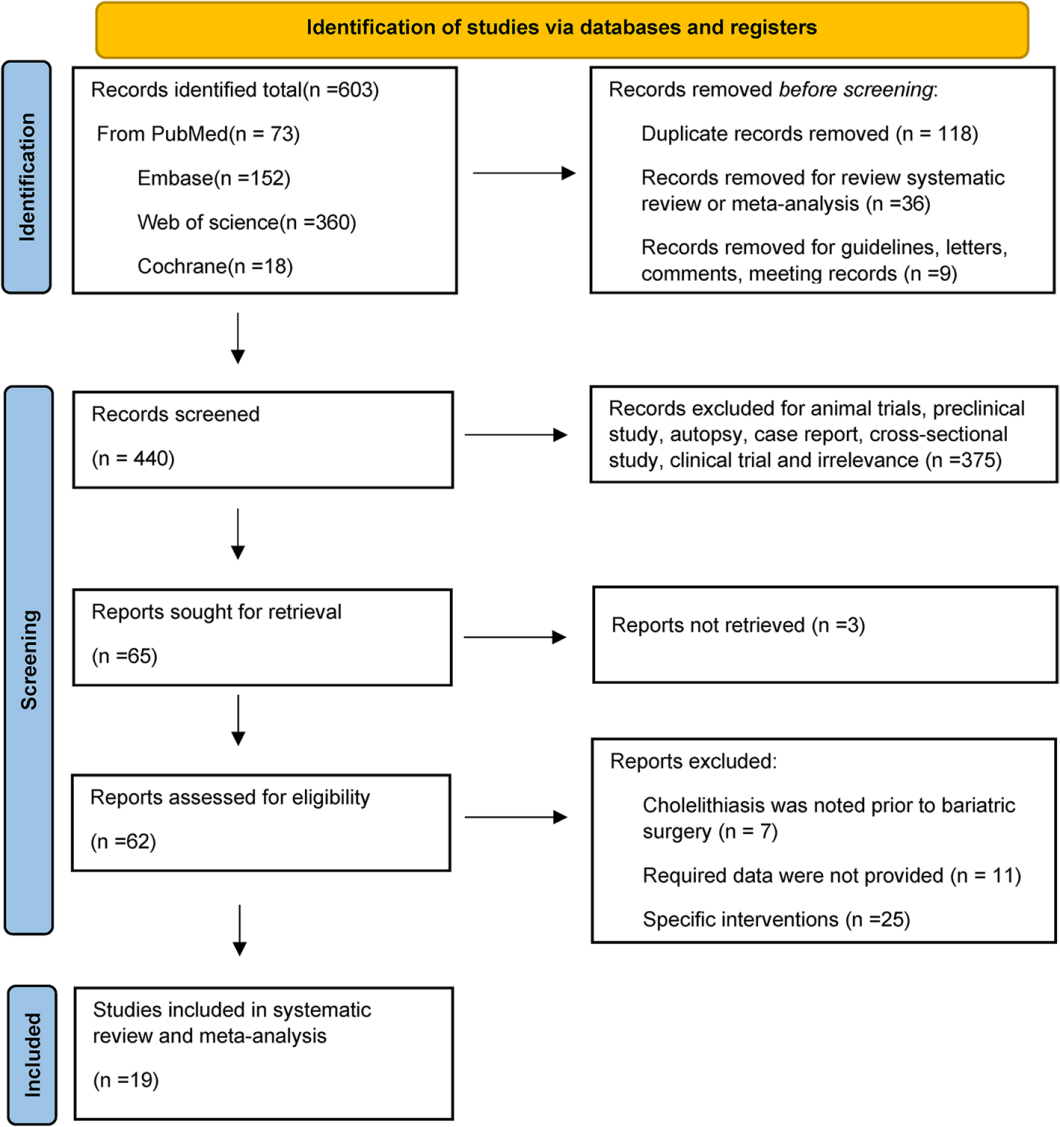
Screening process

In the literature search, a total of 603 studies were retrieved, and they were screened according to the preestablished inclusion criteria. A total of 19 studies were finally included in the meta-analysis, as shown in Fig. 1.

### Study characteristics

A total of 20,553 patients were included in this meta-analysis, including 5169 male patients and 15,384 female patients. The basic information of the patients in each study is shown in Table 1.

**Table 1:** Basic information of the included articles and patients

NO	author	year	country	age (mean ± SD)	sex (male/female)	Preoperative BMI (kg/m2)	Preoperative weight (kg)	follow up (year)	surgery type	quality score
1	Mohammed A. Aldriweesh	2020	Saudi Arabia	36.87 ± 11.44	189/301	46.15 ± 6.94	123.31 ± 21.89	2	SG RYGB	8
2	Faisal A. Alsaif	2019	Saudi Arabia	31.1 ± 14(with CL) 34.7 ± 11.9(without CL)	313/414	46 ± 8.7(with CL) 45 ± 10.3(without CL)	123.1 ± 28.4	2	LSG	7
3	Ainhoa Andre’s-Imaz	2020	Spain	44.92(all) 40.83 ± 12.57(with CL) 44.35 ± 11.9(without CL)	88/192	46.24 ± 7.1(with CL) 46.02 ± 7.73(without CL)	\	6.47(mean)	SG RYGB	8
4	Muriel Coupaye, M.D.	2014	France	40.4 ± 11.9 (with CL) 41.9 ± 10.8(without CL)	15/145	46.2 ± 7.5 (with CL) 44.3 ± 5.3(without CL)	125.1 ± 22.5 (with CL) 119.7 ± 17.3(without CL)	4	SG RYGB	8
5	Hernán M. Guzmán	2019	Chile	37.8 ± 10.5	80/96	37.5	\	1	SG GB RYGB	7
6	Sylke Haal	2022	Netherlands	44.8 ± 11.3	150/519	40.1 ± 4.8	115.7 ± 18.3	2	SG RYGB	8
7	Sylke Haal	2019	Netherlands	39.8 ± 10.6(with CL) 43.8 ± 10.5(without CL)	109/590	43.4 ± 5.1 (with CL) 43.4 ± 5.0 (without CL)	\	2	RYGB	8
8	Rosalie M. Kiewiet, MD	2006	Netherlands	43.5 ± 11.3(with CL) 41.4 ± 8.0(without CL)	10/88	45.0 ± 4.6(with CL) 44.0 ± 6.1(without CL)	131.8 ± 17.0(with CL) 129.4 ± 17.7(without CL)	4.68(mean)	AGB	6
9	Mehmet Celal Kızılkaya	2020	Turkey	36.43 ± 9.52(all) 36.28 ± 9.58(with CL) 36.87 ± 9.44(without CL)	34/151	44.16 ± 5.09(all) 45.31 ± 4.77(with CL) 40.76 ± 4.45(without CL)	\	2	SG RYGB	6
10	Vicky Ka Ming Li	2009	USA	39.9(with CL) 43.0(without CL)	142/444	46.5(with CL) 47.7(without CL)	\	2	LSG LAG RYGB	7
11	Wuttiporn Manatsathit, MD	2016	USA	44.9 ± 12.0 (with CL) 44.0 ± 12.06 (without CL)	20/76	49.5 ± 9.6 (with CL) 48.6 ± 6.2 (without CL)	136.98 ± 30.62(with CL) 133.81 ± 26.67 (without CL)	2	SG RYGB	7
12	Andreas Melmer	2015	Austria	55.3 ± 10.5	28/91	\	118.2 ± 22.9 (with CL) 119.9 ± 14.9 (without CL)	10.42(mean)	SG LAGB	7
13	Rena C. Moon, M.D.	2013	USA	44.9 ± 12.0 (LSG) 44.0 ± 12.06 (LRYGB)	120/362	46.0 ± 7.9(LSG) 47.1 ± 7.7 (LRYGB)	\	2	LSG LRYGB	7
14	Rachid Nagem	2012	Brazil	40.6 (with CL) 42.2 (without CL)	4/34	46.2 (with CL) 48.9 (without CL)	\	3	RYGB	7
15	Sabri Özdaş	2019	Turkey	37.1 ± 10.8(all) 42.7 ± 10.3(with CL) 35.6 ± 10.7(without CL)	35/95	46.2 ± 3.8(all) 46.1 ± 3.5(with CL) 45.6 ± 3.7(without CL)	\	1	LSG	7
16	Sidney Pinheiro-Júnior	2012	Brazil	46.6 ± 11.2 (with CL) 40.6 ± 9.7 (without CL)	41/179	50.2 ± 12.1 (with CL) 47.9 ± 14.2 (without CL)	\	0.67(mean)	RYGB	7
17	M. Plecka Östlund	2012	Sweden	40	3436/10007	\	\	7.39(mean)	GB	8
18	Midhat Abu Sneineh	2020	Israel	48 ± 19	123/436	\	\	1	LSG LAG RYGB	7
19	Victor B. Tsirline, M.D.	2014	USA	39.9 ± 9.3 (with CL) 42.8 ± 9.9 (without CL)	232/1164	45.2 ± 5.3 (with CL) 44.6 ± 5.5(without CL)	\	4	LSG LAG RYGB	7

*CL* cholelithiasis; *GB* gastric bypass; *SG* sleeve gastrectomy; *LSG* laparoscopic sleeve gastrectomy; *RYGB* Roux-en-Y gastric bypass; *LRYGB* laparoscopic Roux-en-Y gastric bypass; *AGB* adjustable gastric banding; *LAGB* laparoscopic adjustable gastric banding; *BMI* body mass index

### Quality assessment

All the studies included in the meta-analysis used the NOS scale to evaluate the literature quality. All the articles included scored above 6 and were considered high-quality articles. The detailed evaluation results are shown in Table 2.

**Table 2:** Literature quality assessment according to NOS

NO	Author	Year	Study type	Selection	Comparability	Exposure	Outcome	Total
1	Mohammed A. Aldriweesh	2020	cohort study	★★★★	★	\	★★★	8
2	Faisal A. Alsaif	2019	cohort study	★★★	★	\	★★★	7
3	Ainhoa Andre’s-Imaz	2020	case control study	★★★★	★	★★★	\	8
4	Muriel Coupaye, M.D.	2014	cohort study	★★★★	★	\	★★★	8
5	Hernán M. Guzmán	2019	cohort study	★★★	★	\	★★★	7
6	Sylke Haal	2022	cohort study	★★★★	★	\	★★★	8
7	Sylke Haal	2019	case control study	★★★★	★	★★★	\	8
8	Rosalie M. Kiewiet, MD	2006	cohort study	★★★	★	\	★★	6
9	Mehmet Celal Kızılkaya	2020	cohort study	★★★	★	\	★★	6
10	Vicky Ka Ming Li	2009	case control study	★★★	★	★★★	\	7
11	Wuttiporn Manatsathit, MD	2016	cohort study	★★★★	★	\	★★	7
12	Andreas Melmer	2015	cohort study	★★★	★	\	★★★	7
13	Rena C. Moon, M.D.	2013	case control study	★★★	★	★★★	\	7
14	Rachid Nagem	2012	cohort study	★★★	★	\	★★★	7
15	Sabri Özdaş	2019	cohort study	★★★	★	\	★★★	7
16	Sidney Pinheiro-Júnior	2012	case control study	★★★	★	★★★	\	7
17	M. Plecka Östlund	2012	cohort study	★★★★	★	\	★★★	8
18	Midhat Abu Sneineh	2020	cohort study	★★★★	★	\	★★	7
19	Victor B. Tsirline, M.D.	2014	cohort study	★★★	★	\	★★★	7

### Meta-analysis

#### The influence of the patient’s basic condition on the occurrence of cholelithiasis after bariatric surgery

##### Sex

**Fig. 2:**
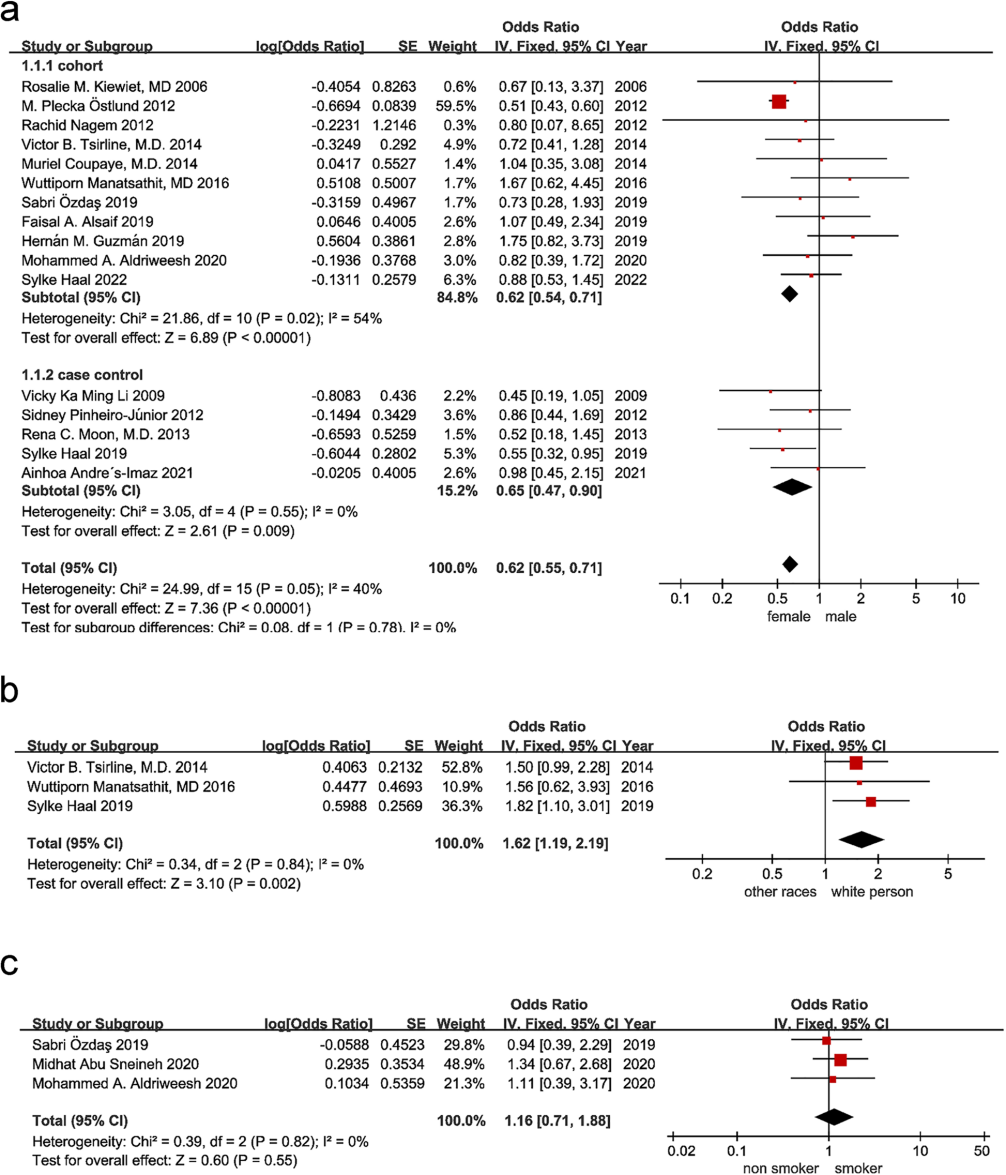
Forest plots of sex, race and smoking. (**a**) sex; (**b**) race; (**c**) smoking

Defining men as exposed and women as nonexposed, 16 articles [6, 16–30] evaluated whether sex is a risk factor for cholelithiasis after bariatric surgery. There was slight heterogeneity among the studies (I^2^ = 40%, *P* = 0.05), so a fixed-effect model was used for meta-analysis. The results are shown in Fig. 2. Female sex was a risk factor for cholelithiasis after bariatric surgery [OR = 0.62, 95% CI (0.55, 0.71), *P* < 0.00001]. In addition, the sex sensitivity analysis and publication bias test can be found in the supplementary materials, and the results show that the combined effect size of sex was relatively stable and had no publication bias (*P* > 0.05).

##### Race

Caucasians were defined as exposed, and other races were defined as nonexposed. Three articles evaluated whether race is a risk factor for cholelithiasis after bariatric surgery [20, 24, 30] There was no heterogeneity among the studies (I^2^ = 0%, *P* = 0.84), so a fixed-effects model was used for the meta-analysis. The results showed that white race was a risk factor for cholelithiasis after bariatric surgery [OR = 1.62, 95% CI (1.19, 2.19), *P* = 0.002].

##### Smoking

A meta-analysis was performed to determine whether smoking affects cholelithiasis after bariatric surgery and defined smoking as exposure and nonsmoking as nonexposure. A total of 3 articles [16, 27, 31] were included. There was no heterogeneity among the studies (I^2^ = 0%, *P* = 0.82), so a fixed-effects model was used for the meta-analysis. As shown in Fig. 2, smoking was not a risk factor for cholelithiasis after bariatric surgery [OR = 1.16, 95% CI (0.71,1.88), *P* = 0.55].

#### Exploration of the relationship between surgical conditions and the occurrence of cholelithiasis

##### Surgical procedure

**Fig. 3:**
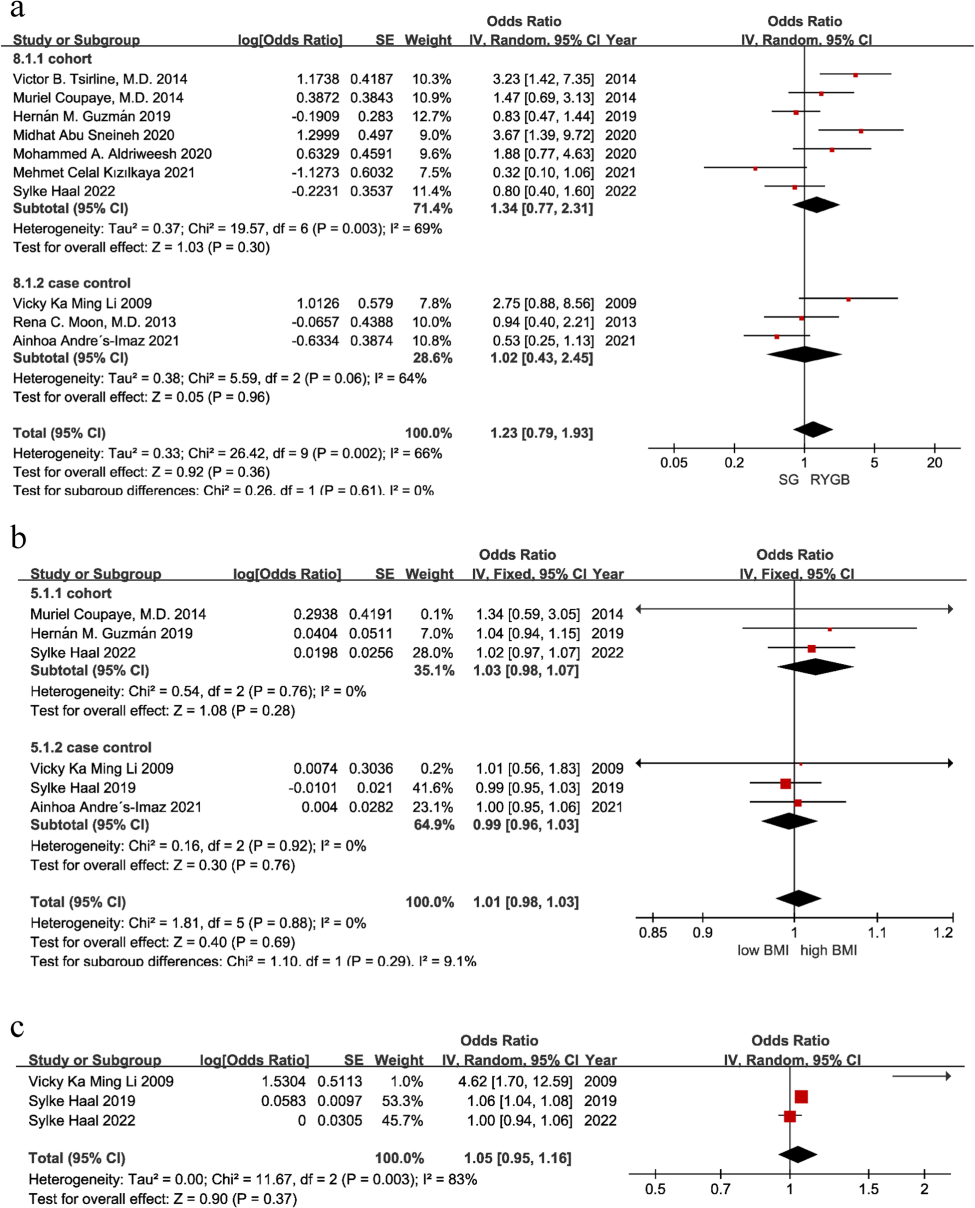
Forest plots of the procedure, initial BMI and %TWL. (**a**) Procedure; (**b**) initial BMI; (**c**) %TWL

This research examined two surgical procedures, RYGB and SG, and defined RYGB as exposure and SG as nonexposure. A total of 10 articles [16–20, 23, 25, 30–32] investigated whether the surgical procedure is a risk factor for cholelithiasis after bariatric surgery. There was moderate heterogeneity (I^2^ = 66%, *P* = 0.002), so a random-effect model was used for the meta-analysis. As shown in Fig. 3, RYGB was not a risk factor for cholelithiasis after bariatric surgery [OR = 1.23, 95% CI (0.79, 1.93), *P* = 0.36]. Due to the moderate heterogeneity among the studies, the literature was reviewed and eliminated one by one, and the heterogeneity was not significantly reduced, so sensitivity analysis and publication bias tests of the surgical method were carried out. The results were stable, and the funnel plot suggested that there may be publication bias. Therefore, Egger’s test was performed to determine whether there was publication bias. The Egger test showed *P* = 0.387, indicating no publication bias.

##### Preoperative BMI

Many original studies have explored preoperative BMI as a risk factor. Except for Muriel Coupaye’s study [18], in which BMI > 50 was defined as exposure, and Vicky Ka Ming Li′s article [23], in which BMI > 45 was defined as exposure. Other articles did not describe the specific definition of high BMI, but we collected its OR value. High BMI was defined as exposure, and low BMI was defined as nonexposure. A total of 6 articles [17–21, 23] were included in the meta-analysis. There was no heterogeneity among the studies (I^2^ = 0%, *P* = 0.88), so a fixed-effect model was used for pooling. As shown in Fig. 3, preoperative BMI was not a risk factor for cholelithiasis after bariatric surgery [OR = 1.01, 95% CI (0.98, 1.03), *P* = 0.69].

##### Weight loss after surgery

Many studies have explored the influence of postoperative total weight loss (%TWL), total BMI reduction (%TBMIL), excess weight loss (%EWL), and excess BMI loss (%EBMIL) after bariatric surgery. However, due to the insufficient number of studies exploring weight loss as a risk factor, only %TWL data were extracted for meta-analysis during data extraction. There was high heterogeneity among studies (I^2^ = 83%, *P* = 0.003), so a random-effects model was used for pooling. As shown in Fig. 3, the level of %TWL was not a risk factor for cholelithiasis after bariatric surgery [OR = 1.05, 95% CI (0.95, 1.16), *P* = 0.37]. There was high heterogeneity among studies, and the number of studies was too small to conduct subgroup analysis. After reviewing the literature, it was found that the article by Vicky Ka Ming Li [23] divided patients into %TWL > 25% and %TWL < 25% according to %TWL. The two studies published by Sylke Haal in 2019 and 2022 [20, 21] calculated ORs by regression analysis, so exposure was not defined, which may be the cause of heterogeneity. In addition, the study of Sylke Haal 2019 [21] included only the RYGB procedure, while the study of Sylke Haal 2022 [20] included both RYGB and SG procedures, and the study of Vicky Ka Ming Li also included the AGB procedure, which may also be a source of heterogeneity.

#### Influence of underlying diseases on the occurrence of postoperative cholelithiasis

##### Hypertension

**Fig. 4:**
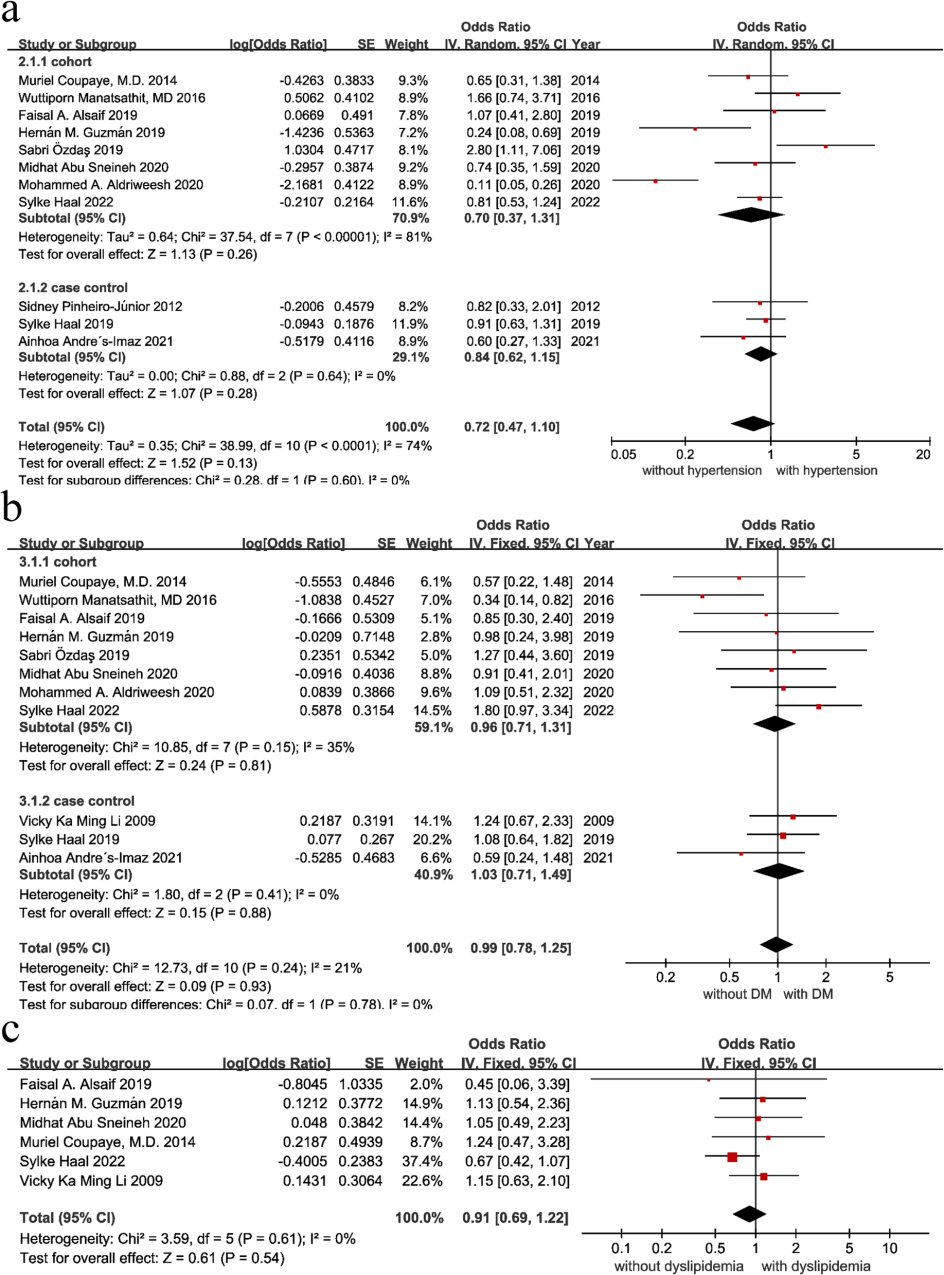
Forest plots of hypertension, DM and dyslipidemia. (**a**) hypertension; (**b**) DM; (**c**) dyslipidemia

Hypertension was defined as exposure and nonexposure without hypertension. Eleven articles in total [6, 16–21, 24, 27, 28, 31] investigated the effect of hypertension on cholelithiasis after bariatric surgery. With moderate heterogeneity among studies (I^2^ = 74%, *P* < 0.0001), a random-effect model was used for meta-analysis. As shown in Fig. 4, the results showed that hypertension was neither a risk factor nor a protective factor for cholelithiasis after bariatric surgery [OR = 0.72, 95% CI (0.47, 1.10), *P* = 0.13]. Due to the moderate heterogeneity among the studies, the literature was reviewed and checked one by one. The article by Mohammed A. Aldriweesh was removed, and the meta-analysis was performed again. The heterogeneity was reduced to I^2^ = 45%, *P* = 0.06 [OR = 0.87, 95% CI (0.64,1.17), *P* = 0.35]. If the article by Mohammed A. Aldriweesh and the article by Sabri Özdaş were removed and the meta-analysis was performed again, the heterogeneity was reduced to I^2^ = 18%, *P* = 0.28 [OR = 0.80, 95% CI (0.63, 1.13), *P* = 0.08]. Therefore, a sensitivity analysis and publication bias test of hypertension were carried out. The results of the sensitivity analysis showed that the results were stable after excluding the studies one by one, and the funnel plot suggested that there might be publication bias. Therefore, Egger’s test was performed to determine whether there was publication bias. The Egger test showed that *P* = 0.718, with no publication bias.

##### Diabetes

To explore the effect of diabetes on postoperative cholelithiasis, diabetes was defined as exposure, and the absence of diabetes was defined as nonexposure. A total of 11 articles [6, 16–21, 23, 24, 27, 31] were included, with no heterogeneity among the studies (I^2^ = 21%, *P* = 0.24), so a fixed-effect model was used for the meta-analysis. As shown in Fig. 4, the results show that diabetes is neither a risk factor nor a protective factor for postoperative cholelithiasis [OR = 0.99, 95% CI (0.78, 1.25), *P* = 0.93]. In addition, the sensitivity analysis and publication bias test of diabetes supporting the findings. The results of the sensitivity analysis show that the combined effect size of diabetes was stable, and the funnel plot suggests that there may be publication bias. Therefore, Egger’s test was performed to determine whether there was publication bias. The Egger test determined that *P* = 0.141, with no publication bias.

##### Dyslipidemia

Dyslipidemia was defined as exposure, and no hypertension was defined as nonexposure [6, 18–20, 23, 31]. A total of 6 articles investigated the effect of dyslipidemia on cholelithiasis after bariatric surgery, with no heterogeneity among the studies (I^2^ = 0%, *P* = 0.61), so a fixed-effects model was used for the meta-analysis. As shown in Fig. 4, dyslipidemia was neither a risk factor nor a protective factor for cholelithiasis after bariatric surgery [OR = 0.91, 95% CI (0.69,1.22), *P* = 0.54].

(The results of sensitivity analysis and funnel diagram of the above contents can be found in Additional files 2 and 3)

In addition, we conducted subgroup analysis (classified by research type) on BMI, diabetes, hypertension, sex and surgical type. The results are the same as above.

## Discussion

There are many original studies exploring the risk factors for cholelithiasis after bariatric surgery, but regarding conclusions, in addition to the current evidence suggesting that UDCA can prevent the occurrence of postoperative cholelithiasis [33–36], it is difficult to reach consensus on other factors. The results of this study show that Caucasians and women are risk factors for cholelithiasis after bariatric surgery; surgical procedure, BMI, weight loss, hypertension, diabetes mellitus, dyslipidemia, and smoking are not risk factors for concurrent postoperative cholelithiasis.

The results of the present study show that Caucasian ethnicity is one of the risk factors, but data from East Asian populations were not taken into account in our meta-analysis due to the lack of original studies reporting from East Asian populations. More studies are needed in the future to provide a more comprehensive discussion of whether race is a risk factor.

The surgical procedures in this meta-analysis included RYGB and SG because these two are highly practical, and other procedures, especially AGB, have serious adverse reactions [37], so they are rarely used. RYGB is a representative and highly efficient procedure in bariatric surgery [38]. SG is currently the most commonly used surgical procedure in the world, and its adverse event rate is lower than that of RYGB [39]. Regarding whether the different surgical methods will cause changes in the incidence of cholelithiasis, the current controversy is whether the incidence of cholelithiasis after RYGB is higher than that of other procedures [31]. Many studies have suggested that cholecystectomy should be performed before or at the same time as RYGB; otherwise, the patient will be more prone to postoperative cholelithiasis [40, 41]. Although two studies in the included studies suggested that RYGB increased the risk of postoperative cholelithiasis, the results of this meta-analysis show that RYGB does not increase the risk of postoperative cholelithiasis. A previous meta-analysis showed that the incidence of cholelithiasis after RYGB was higher than that after SG [42] We read this meta-analysis carefully and found that the reason for the difference between our conclusions may be that we excluded studies using UCDA intervention, whereas Wan et al. did not control for that possible bias. In conclusion, it still needs to be decided according to the patients’ condition as well as whether cholecystectomy should be performed before or during the operation. For patients without postoperative cholelithiasis, the same management measures can be used as in the nonobese population. At least 60% of patients will not be complicated with cholelithiasis after surgery, so cholecystectomy is unnecessary and prolongs the hospital stay [11, 43].

People with obesity have a higher risk of developing cholelithiasis than nonobese people, and bariatric surgery, despite inducing rapid weight loss, can increase the chance of developing cholelithiasis [36, 44]. The RYGB procedure, in particular, has been linked to an increased risk of cholelithiasis because of its ability to rapidly lose more weight [45]. This meta-analysis shows that initial BMI is not a risk factor. This is inconsistent with the conclusions drawn from long-term observations [41] and may be due to the rapid decline in short-term BMI caused by bariatric surgery and insufficient follow-up time [41, 46].

First, two authors independently extracted data with %TWL,% TBMIL, %EWL, and %EBMIL as risk factors, but the original research data were too small to support the meta-analysis. In our meta-analysis, %TWL was not a risk factor, although two original studies [21, 23] suggested that excessive weight loss may be a risk factor, consistent with current knowledge. For other factors that measure the proportion of weight loss, we conducted a systematic review of the literature. Andres-Imaz [17] indicated that %EWL was a risk factor [OR = 1.03, 95% CI (1.01, 1.05)], and the study by Guzmán [19] suggested that %EBMIL was not a risk factor [OR = 0.99, 95% CI (0.97, 1.00)], but this was based on a single-center cohort study. In addition, multiple studies by Aldriweesh, Faisal et al. [6, 16, 18, 22, 24] showed that there was no statistically significant difference in the %TWL data of patients with postoperative cholelithiasis and those without postoperative cholelithiasis. Aldriweesh and Manatsathit [16, 24] found no significant difference in %EBMIL data between patients with postoperative cholelithiasis and those without postoperative cholelithiasis. Coupaye and Tsirline [18, 30] found no statistically significant difference in %EWL between postoperative cholelithiasis and no postoperative cholelithiasis within six months. Andreas Andreas [17] also found no statistically significant difference in %EWL at up to ten years of follow-up. A randomized controlled trial conducted by Ahmed Talha [47] showed that higher %EWL in short-term follow-up was the cause of postoperative cholelithiasis. However, due to the difficulty of definition and the difficulty of controlling confounding factors, studies using these factors as risk factors need to be carefully designed [45]. In addition, the heterogeneity of this research in weight loss was too high, and no source of heterogeneity was found, so the conclusions must be interpreted cautiously, and more research is needed to confirm the findings.

The pathophysiological process of gallstone formation involves abnormal cholesterol metabolism, and dyslipidemia is closely related to it. High levels of serum triglycerides and low-density lipoprotein have been reported by scholars as risk factors for gallstone formation [48]. In addition, many studies have found that preoperative dyslipidemia is an independent risk factor for gallstone formation [34, 49]. However, our meta-analysis shows that postoperative dyslipidemia does not appear to increase the risk of cholelithiasis.

Whether hypertension is a factor in postoperative cholelithiasis has been controversial. Aldriweesh and Guzmán suggested that hypertension is a protective factor [16, 19], but Sabri Özdaş’s study concluded that hypertension is a risk factor [27]. A cross-sectional study also showed that the severity of hypertension is closely related to the formation of gallstones, and the higher the severity of hypertension is, the higher the risk of developing cholelithiasis [50]. However, whether or not articles with high heterogeneity and poor sensitivity were excluded from this meta-analysis, the conclusion remains unchanged; that is, hypertension is neither a risk factor nor a protective factor.

Diabetes is a risk factor for the formation of gallstones larger than 1 cm [51] and an independent risk factor for cholecystectomy [44]. In a meta-analysis of stone association analyses, multiple associations were found between diabetes, hypertension, gallstones, and kidney stones [52]. According to our results, neither diabetes nor hypertension appear to increase the risk of postoperative cholelithiasis. However, bariatric surgery itself has certain curative effects on these underlying diseases [37]. Especially in the context of diabetes, many RCTs have demonstrated that bariatric surgery appears to be better than medical treatment for diabetes [37]. In the short term (1–2 years), the control effect of hypertension and dyslipidemia is better than that of drugs, or the conventional dosage can be reduced [53, 54]. For this reason, whether the definitions of postoperative diabetes, dyslipidemia, and hypertension should be different from conventional ones and whether patients with preoperative symptoms but negative postoperative symptoms should be included in the study may cause biases in our findings. In this regard, more well-designed studies are needed to support these conclusions.

Finally, most studies controlled for age at baseline and were therefore not included in the meta-analysis. There were also many studies that explored sleep respiratory distress syndrome, pneumonia, renal failure, and liver cirrhosis as risk factors, but the number of studies was too small to be included in the meta-analysis.

### Strengths and limitations

This meta-analysis is the first article to systematically summarize and scientifically describe risk factors for cholelithiasis after bariatric surgery. A total of 19 articles with 20,553 patients were included, covering all possible risk factors for meta-analysis.

This study has the following limitations: there was heterogeneity in the proportion of postoperative weight loss, and the source of heterogeneity could not be identified based on current methods; the definitions of risk factors in many studies were not completely accurate, such as the definition of postoperative hypertension. But in this study, clinical diagnostic criteria were used to assign the pooled default definitions; the number of original studies was insufficient, and the risk factors included were not comprehensive due to incomplete data. Only three articles are included in TWL analysis. The above shortcomings need to be carefully considered when interpreting the conclusions of this study.

## Conclusions

In conclusion, Caucasian ethnicity and female sex are risk factors for cholelithiasis after bariatric surgery. For women and Caucasians, applying corresponding protective treatment after bariatric surgery is of higher priority in clinical decision making, such as UCDA. Surgical procedures, rapid weight loss, postoperative underlying diseases, and poor habits in the context of the occurrence of postoperative cholelithiasis still need more research for verification. There is no need to conduct protective methods routinely for these patients based on the current research conclusion.

## Supplementary Information

**Additional file 1.** Retrieval strategy.

**Additional file 2.** Sensitivity analysis.

**Additional file 3.** Funnel diagram.

**Additional file 4.** PRISMA checklist.

**Additional file 5. **publication bias test. (a) DM; (b) Dyslipidemia; (c) Gender; (d) Hypertension; (e) Surgical procedure.

## Data Availability

All data generated or analyzed during this study are included in this published article and its supplementary information files.
