# Supplementary material for: Incidence and risk factors for cholelithiasis after bariatric surgery: a systematic review and meta-analysis
Source: Lipids Health Dis. 2023 Jan 14;22:5. doi: 10.1186/s12944-023-01774-7 (PMC9840335; doi:10.1186/s12944-023-01774-7)
Supplement: Supplementary file 1 — Additional file 1. Retrieval strategy. [file 12944_2023_1774_MOESM1_ESM.pdf]

Additional file 1. Retrieval strategy

PubMed：73 studies
MESH：Bariatric Surgery　　Gallstones（Cholelithiasis Cholecystolithiasis　Calculi）
Risk Factors
Entry terms：Surgeries, Bariatric
Surgery, Bariatric
Metabolic Surgery
Metabolic Surgeries
Surgeries, Metabolic
Surgery, Metabolic
Bariatric Surgical Procedures
Bariatric Surgical Procedure
Procedure, Bariatric Surgical
Procedures, Bariatric Surgical
Surgical Procedure, Bariatric
Surgical Procedures, Bariatric
Bariatric Surgeries
Stomach Stapling
Stapling, Stomach

Gallstone
Gall Stones
Biliary Calculi
Calculi, Biliary
Gall Stone
Common Bile Duct Calculi
Biliary Calculi, Common Bile Duct
Gallstones, Common Bile Duct
Common Bile Duct Gall Stone
Common Bile Duct Gallstones
Gall Stones, Common Bile Duct
Common Bile Duct Gallstone
Common Bile Duct Gall Stones

Factor, Risk
Risk Factor
Social Risk Factors
Factor, Social Risk
Factors, Social Risk
Risk Factor, Social
Risk Factors, Social
Social Risk Factor
Health Correlates

Correlates, Health
Population at Risk
Populations at Risk
Risk Scores
Risk Score
Score, Risk
Risk Factor Scores
Risk Factor Score
Score, Risk Factor

((("Bariatric Surgery"[Mesh]) OR (((((((((((((((Bariatric Surgery[Title/Abstract]) OR (Surgeries, Bariatric[Title/Abstract])) OR (Surgery, Bariatric[Title/Abstract])) OR (Metabolic Surgery[Title/Abstract])) OR (Metabolic Surgeries[Title/Abstract])) OR (Surgeries, Metabolic[Title/Abstract])) OR (Surgery, Metabolic[Title/Abstract])) OR (Bariatric Surgical Procedures[Title/Abstract])) OR (Bariatric Surgical Procedure[Title/Abstract])) OR (Procedure, Bariatric Surgical[Title/Abstract])) OR (Procedures, Bariatric Surgical[Title/Abstract])) OR (Surgical Procedure, Bariatric[Title/Abstract])) OR (Surgical Procedures, Bariatric[Title/Abstract])) OR (Bariatric Surgeries[Title/Abstract])) OR (Stomach Stapling[Title/Abstract])) OR (Stapling, Stomach[Title/Abstract]))) AND (("Gallstones"[Mesh]) OR (((((((((((((Gallstones[Title/Abstract]) OR (Gallstone[Title/Abstract])) OR (Gall Stones[Title/Abstract])) OR (Biliary Calculi[Title/Abstract])) OR (Calculi, Biliary[Title/Abstract])) OR (Gall Stone[Title/Abstract])) OR (Common Bile Duct Calculi[Title/Abstract])) OR (Biliary Calculi, Common Bile Duct[Title/Abstract])) OR (Gallstones, Common Bile Duct[Title/Abstract])) OR (Common Bile Duct Gall Stone[Title/Abstract])) OR (Common Bile Duct Gallstones[Title/Abstract])) OR (Gall Stones, Common Bile Duct[Title/Abstract])) OR (Common Bile Duct Gallstone[Title/Abstract])) OR (Common Bile Duct Gall Stones[Title/Abstract])))) AND (("Risk Factors"[Mesh]) OR ((((((((((((((((((Risk Factors[Title/Abstract]) OR (Factor, Risk[Title/Abstract])) OR (Risk Factor[Title/Abstract])) OR (Social Risk Factors[Title/Abstract])) OR (Factor, Social Risk[Title/Abstract])) OR (Factors, Social Risk[Title/Abstract])) OR (Risk Factor, Social[Title/Abstract])) OR (Risk Factors, Social[Title/Abstract])) OR (Social Risk Factor[Title/Abstract])) OR (Health Correlates[Title/Abstract])) OR (Correlates, Health[Title/Abstract])) OR (Population at Risk[Title/Abstract])) OR (Populations at Risk[Title/Abstract])) OR (Risk Scores[Title/Abstract])) OR (Risk Score[Title/Abstract])) OR (Score, Risk[Title/Abstract])) OR (Risk Factor Scores[Title/Abstract])) OR (Risk Factor Score[Title/Abstract])) OR (Score, Risk Factor[Title/Abstract])))

Embase 152studies

Emtree terms：bariatric surgery　gallstone　risk factor

strategy ：('bariatric surgery':ab,ti OR 'surgeries, bariatric':ab,ti OR 'surgery, bariatric':ab,ti OR 'metabolic surgery':ab,ti OR 'metabolic surgeries':ab,ti OR 'surgeries, metabolic':ab,ti OR 'surgery, metabolic':ab,ti OR 'bariatric surgical procedures':ab,ti OR 'bariatric surgical procedure':ab,ti OR 'procedure, bariatric

surgical':ab,ti OR 'procedures, bariatric surgical':ab,ti OR 'surgical procedure, bariatric':ab,ti OR 'surgical procedures, bariatric':ab,ti OR 'bariatric surgeries':ab,ti OR 'stomach stapling':ab,ti OR 'stapling, stomach':ab,ti OR 'bariatric surgery'/mj) AND (gallstone:ab,ti OR 'gall stones':ab,ti OR 'biliary calculi':ab,ti OR 'calculi, biliary':ab,ti OR 'gall stone':ab,ti OR 'common bile duct stone':ab,ti OR 'biliary calculi, common bile duct':ab,ti OR 'gallstones, common bile duct':ab,ti OR 'common bile duct gall stone':ab,ti OR 'common bile duct gallstones':ab,ti OR 'gall stones, common bile duct':ab,ti OR 'common bile duct gallstone':ab,ti OR 'bariatric surgeries':ab,ti OR 'common bile duct gall stones':ab,ti OR 'gallstone'/mj) AND ('risk factor'/exp OR 'factor, risk':ab,ti OR 'risk factor':ab,ti OR 'social risk factors':ab,ti OR 'factor, social risk':ab,ti OR 'factors, social risk':ab,ti OR 'risk factor, social':ab,ti OR 'risk factors, social':ab,ti OR 'social risk factor':ab,ti OR 'health correlates':ab,ti OR 'correlates, health':ab,ti OR 'population at risk':ab,ti OR 'populations at risk':ab,ti OR 'risk scores':ab,ti OR 'risk score':ab,ti OR 'score, risk':ab,ti OR 'risk factor scores':ab,ti OR 'risk factor score':ab,ti OR 'score, risk factor':ab,ti)

Cochrane：same as pubmed

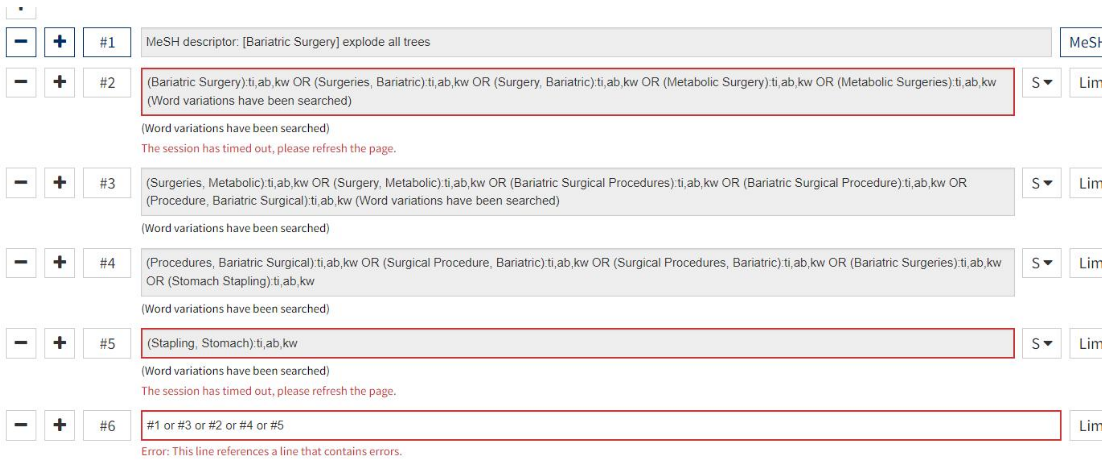

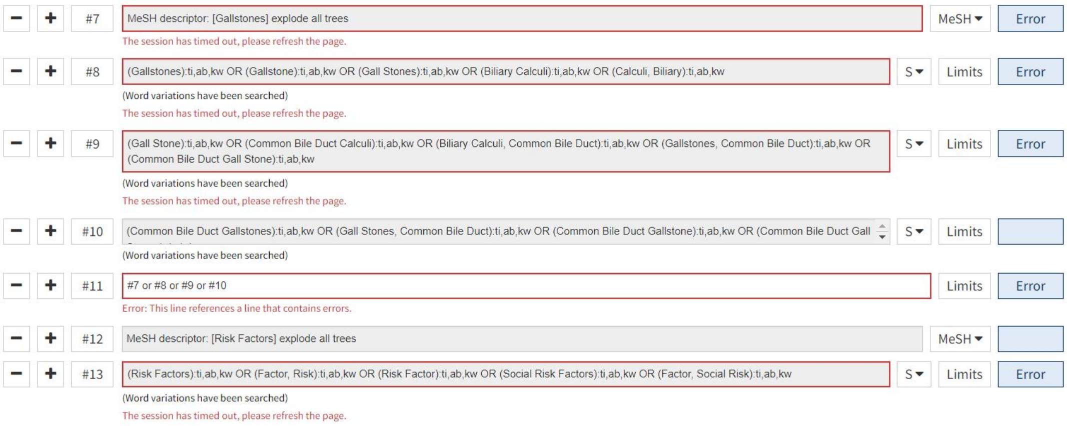

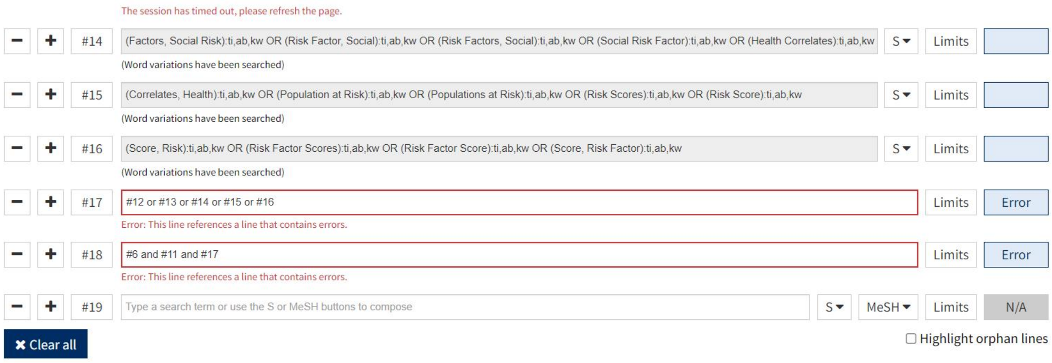

Web Of Science:

1. Bariatric Surgery (Topic) or Surgeries, Bariatric (Topic) or Surgery, Bariatric (Topic) or Metabolic Surgery (Topic) or Metabolic Surgeries (Topic) or Surgeries, Metabolic (Topic) or Surgery, Metabolic (Topic) or Bariatric Surgical Procedures (Topic) or Bariatric Surgical Procedure (Topic) or Procedure, Bariatric Surgical (Topic) or Procedures, Bariatric Surgical (Topic) or Surgical Procedure, Bariatric (Topic) or Surgical Procedures, Bariatric (Topic) or Bariatric Surgeries (Topic) or Stomach Stapling (Topic) or Stapling, Stomach (Topic)

2.
Gallstones (Topic) or Gallstone (Topic) or Gall Stones (Topic) or Biliary Calculi (Topic) or Calculi, Biliary (Topic) or Gall Stone (Topic) or Common Bile Duct Calculi (Topic) or Biliary Calculi, Common Bile Duct (Topic) or Gallstones, Common Bile Duct (Topic) or Common Bile Duct Gall Stone (Topic) or Common Bile Duct Gallstones (Topic) or Gall Stones, Common Bile Duct (Topic) or Common Bile Duct Gallstone (Topic) or Common Bile Duct Gall Stones (Topic)

3. Risk Factors (Topic) or Factor, Risk (Topic) or Risk Factor (Topic) or Social Risk Factors (Topic) or Factor, Social Risk (Topic) or Factors, Social Risk (Topic) or Risk Factor, Social (Topic) or Risk Factors, Social (Topic) or Social Risk Factor (Topic) or Health Correlates (Topic) or Correlates, Health (Topic) or Population at Risk (Topic) or Populations at Risk (Topic) or Risk Scores (Topic) or Risk Score (Topic) or Score, Risk (Topic) or Risk Factor Scores (Topic) or Risk Factor Score (Topic) or Score, Risk Factor (Topic)

#1: Bariatric Surgery (Topic) or Surgeries, Bariatric (Topic) or Surgery, Bariatric (Topic) or Metabolic Surgery (Topic) or Metabolic Surgeries (Topic) or Surgeries, Metabolic (Topic) or Surgery, Metabolic (Topic) or Bariatric Surgical Procedures (Topic) or Bariatric Surgical Procedure (Topic) or Procedure, Bariatric Surgical (Topic) or Procedures, Bariatric Surgical (Topic) or Surgical Procedure, Bariatric (Topic) or Surgical Procedures, Bariatric (Topic) or Bariatric Surgeries (Topic) or Stomach Stapling (Topic) or Stapling, Stomach (Topic)

#2: Gallstones (Topic) or Gallstone (Topic) or Gall Stones (Topic) or Biliary Calculi (Topic) or Calculi, Biliary (Topic) or Gall Stone (Topic) or Common Bile Duct Calculi (Topic) or Biliary Calculi, Common Bile Duct (Topic) or Gallstones, Common Bile Duct (Topic) or Common Bile Duct Gall Stone (Topic) or Common Bile Duct

Gallstones (Topic) or Gall Stones, Common Bile Duct (Topic) or Common Bile Duct Gallstone (Topic) or Common Bile Duct Gall Stones (Topic)
#3: Risk Factors (Topic) or Factor, Risk (Topic) or Risk Factor (Topic) or Social Risk Factors (Topic) or Factor, Social Risk (Topic) or Factors, Social Risk (Topic) or Risk Factor, Social (Topic) or Risk Factors, Social (Topic) or Social Risk Factor (Topic) or Health Correlates (Topic) or Correlates, Health (Topic) or Population at Risk (Topic) or Populations at Risk (Topic) or Risk Scores (Topic) or Risk Score (Topic) or Score, Risk (Topic) or Risk Factor Scores (Topic) or Risk Factor Score (Topic) or Score, Risk Factor (Topic)
#4:#1 AND #2 AND #3
603 in total：PubMed：73，Embase：152，Cochrane：18，WOS：360
