# Supplementary material for: Incidence and risk factors for cholelithiasis after bariatric surgery: a systematic review and meta-analysis
Source: Lipids Health Dis. 2023 Jan 14;22:5. doi: 10.1186/s12944-023-01774-7 (PMC9840335; doi:10.1186/s12944-023-01774-7)
Supplement: Supplementary file 2 — Additional file 2. Sensitivity analysis. [file 12944_2023_1774_MOESM2_ESM.pdf]

Additional file 2. Sensitivity analysis. (a) BMI; (b) DM; (c) dyslipidemia; (d) hypertension; (e) procedure; (f) race; (g) sex.

(a)

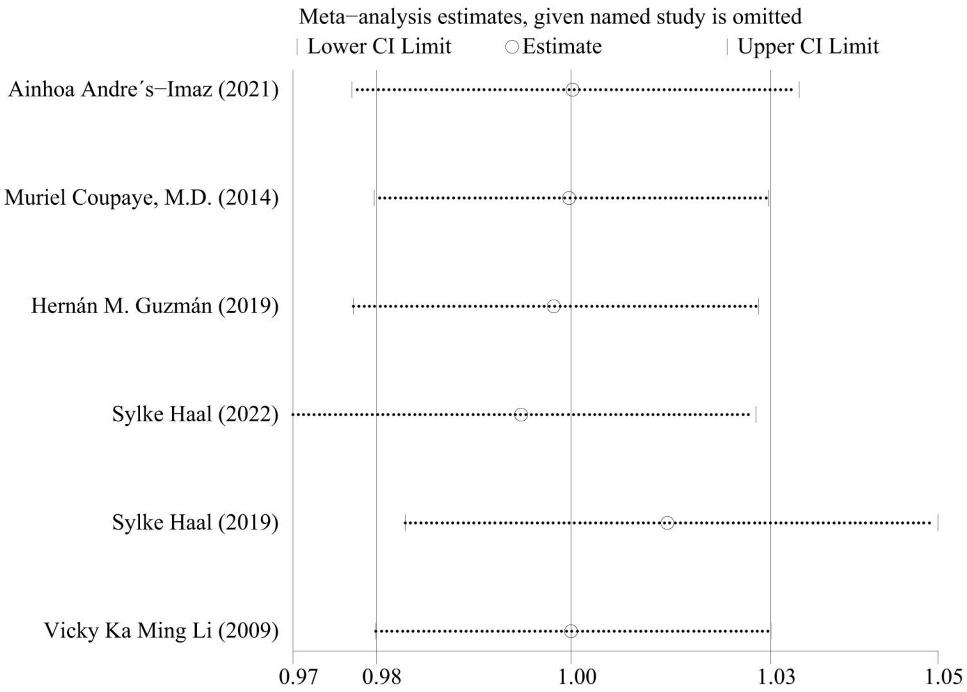

(b)

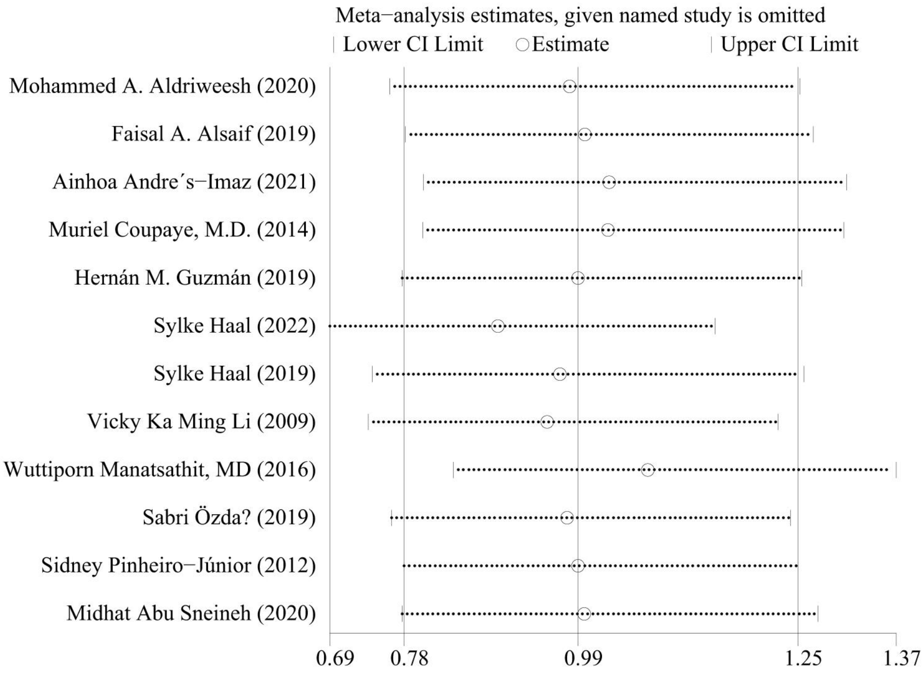
Meta−analysis estimates, given named study is omitted
Lower CI Limit
Estimate
Upper CI Limit
Mohammed A. Aldriweesh (2020)
Faisal A. Alsaif (2019)
Ainhoa Andre´s−Imaz (2021)
Muriel Coupaye, M.D. (2014)
Hernán M. Guzmán (2019)
Sylke Haal (2022)
Sylke Haal (2019)
Vicky Ka Ming Li (2009)
Wuttiporn Manatsathit, MD (2016)
Sabri Özda? (2019)
Sidney Pinheiro−Júnior (2012)
Midhat Abu Sneineh (2020)
0.69
0.78
0.99
1.25
1.37

(c)

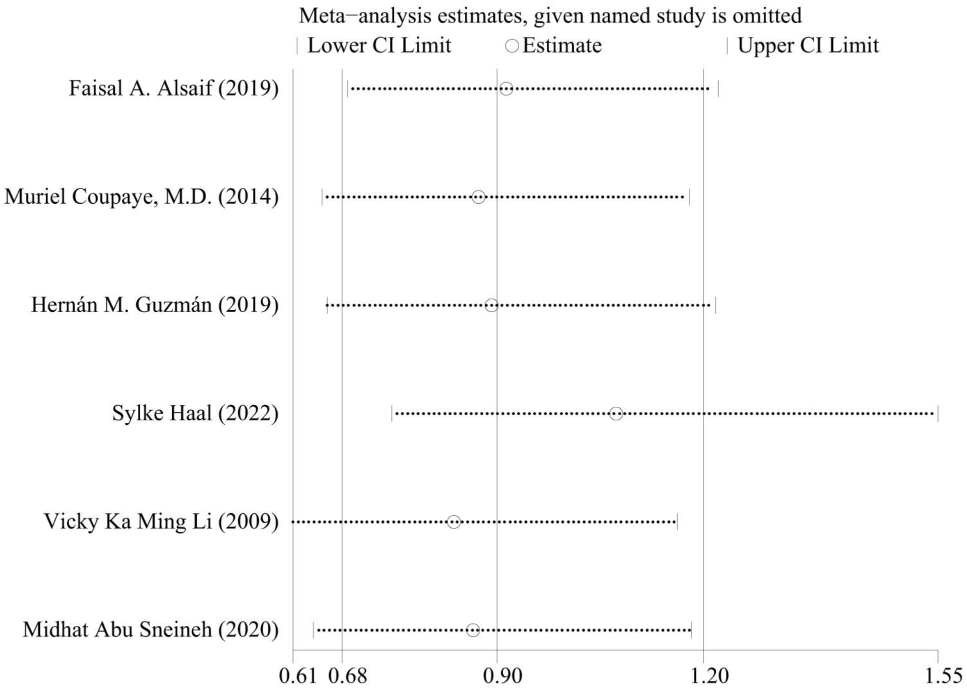
Meta−analysis estimates, given named study is omitted
Lower CI Limit
Estimate
Upper CI Limit
Faisal A. Alsaif (2019)
Muriel Coupaye, M.D. (2014)
Hernán M. Guzmán (2019)
Sylke Haal (2022)
Vicky Ka Ming Li (2009)
Midhat Abu Sneineh (2020)
0.61
0.68
0.90
1.20
1.55

(d)

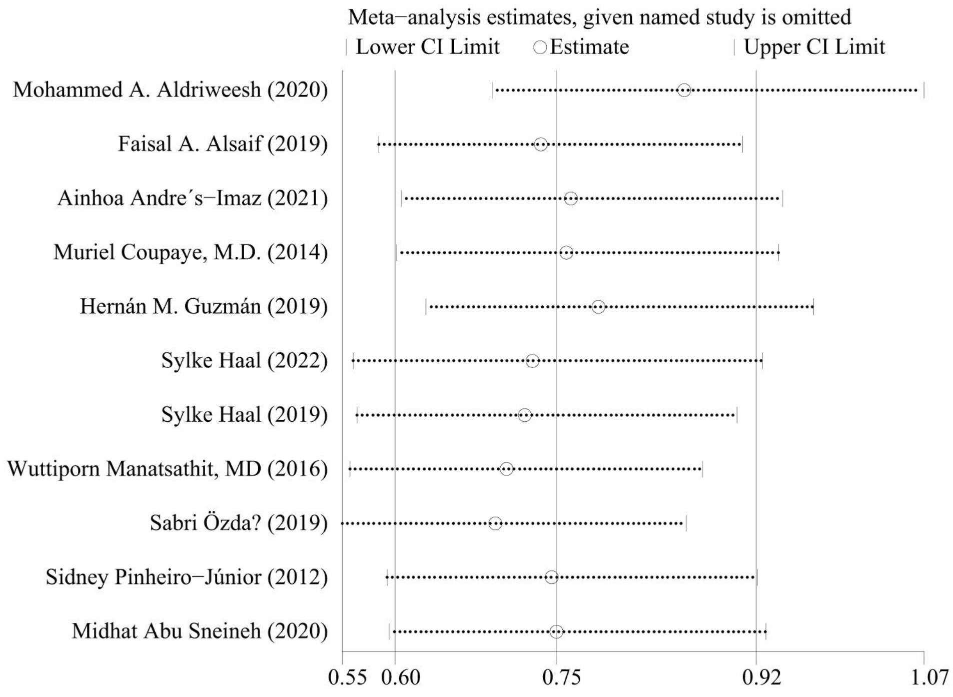

Meta−analysis estimates, given named study is omitted
Lower CI Limit
Estimate
Upper CI Limit
Mohammed A. Aldriweesh (2020)
Faisal A. Alsaif (2019)
Ainhoa Andre´s−Imaz (2021)
Muriel Coupaye, M.D. (2014)
Hernán M. Guzmán (2019)
Sylke Haal (2022)
Sylke Haal (2019)
Wuttiporn Manatsathit, MD (2016)
Sabri Özda? (2019)
Sidney Pinheiro−Júnior (2012)
Midhat Abu Sneineh (2020)
0.55
0.60
0.75
0.92
1.07

(e)

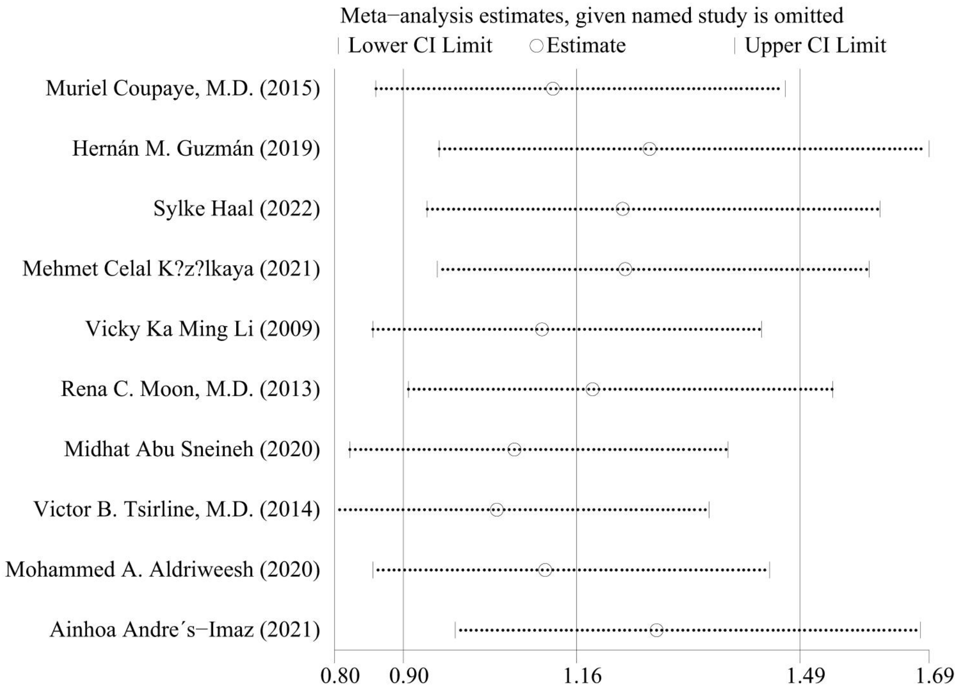

Meta−analysis estimates, given named study is omitted
Lower CI Limit
Estimate
Upper CI Limit
Muriel Coupaye, M.D. (2015)
Hernán M. Guzmán (2019)
Sylke Haal (2022)
Mehmet Celal K?z?lkaya (2021)
Vicky Ka Ming Li (2009)
Rena C. Moon, M.D. (2013)
Midhat Abu Sneineh (2020)
Victor B. Tsirline, M.D. (2014)
Mohammed A. Aldriweesh (2020)
Ainhoa Andre´s−Imaz (2021)
0.80
0.90
1.16
1.49
1.69

(f)

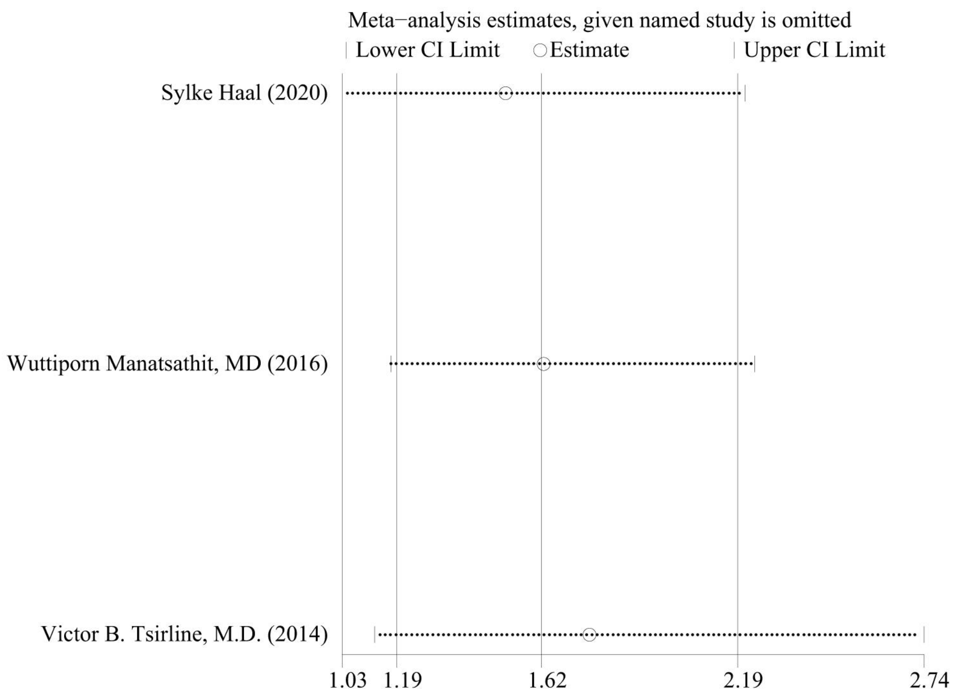
Meta−analysis estimates, given named study is omitted
Lower CI Limit
Estimate
Upper CI Limit
Sylke Haal (2020)
Wuttiporn Manatsathit, MD (2016)
Victor B. Tsirline, M.D. (2014)
1.03
1.19
1.62
2.19
2.74

(g)

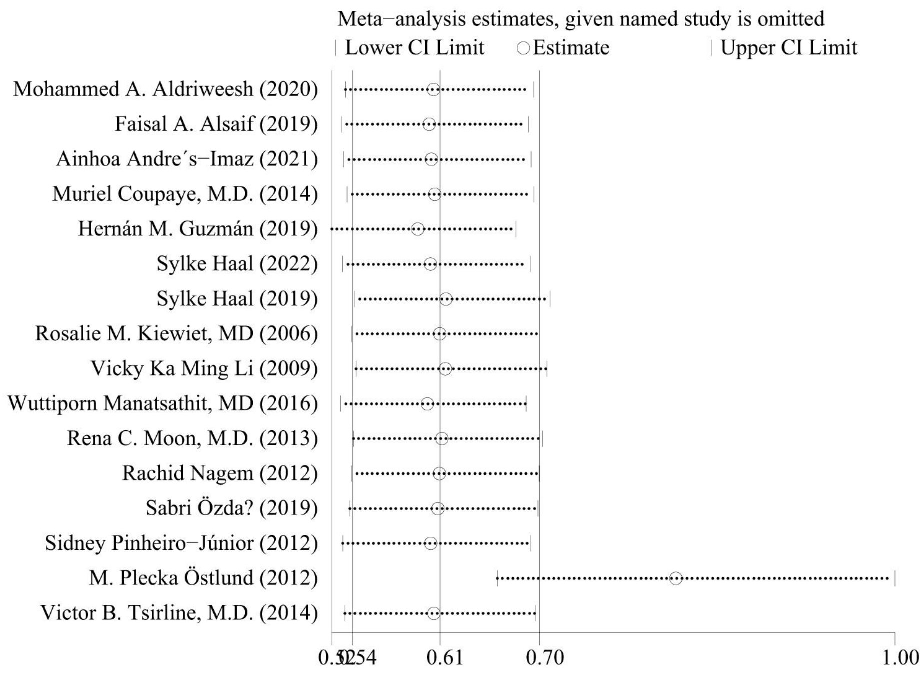
Meta−analysis estimates, given named study is omitted
Lower CI Limit
Estimate
Upper CI Limit
Mohammed A. Aldriweesh (2020)
Faisal A. Alsaif (2019)
Ainhoa Andre´s−Imaz (2021)
Muriel Coupaye, M.D. (2014)
Hernán M. Guzmán (2019)
Sylke Haal (2022)
Sylke Haal (2019)
Rosalie M. Kiewiet, MD (2006)
Vicky Ka Ming Li (2009)
Wuttiporn Manatsathit, MD (2016)
Rena C. Moon, M.D. (2013)
Rachid Nagem (2012)
Sabri Özda? (2019)
Sidney Pinheiro−Júnior (2012)
M. Plecka Östlund (2012)
Victor B. Tsirline, M.D. (2014)
0.61
0.70
1.00
