# Supplementary figures and images for: Incidence and risk factors for cholelithiasis after bariatric surgery: a systematic review and meta-analysis
Source: Lipids Health Dis. 2023 Jan 14;22:5. doi: 10.1186/s12944-023-01774-7 (PMC9840335; doi:10.1186/s12944-023-01774-7)

**Additional file 3. Funnel diagram.** (a) DM; (b) hypertension; (c) procedure; (d) sex.

(a)

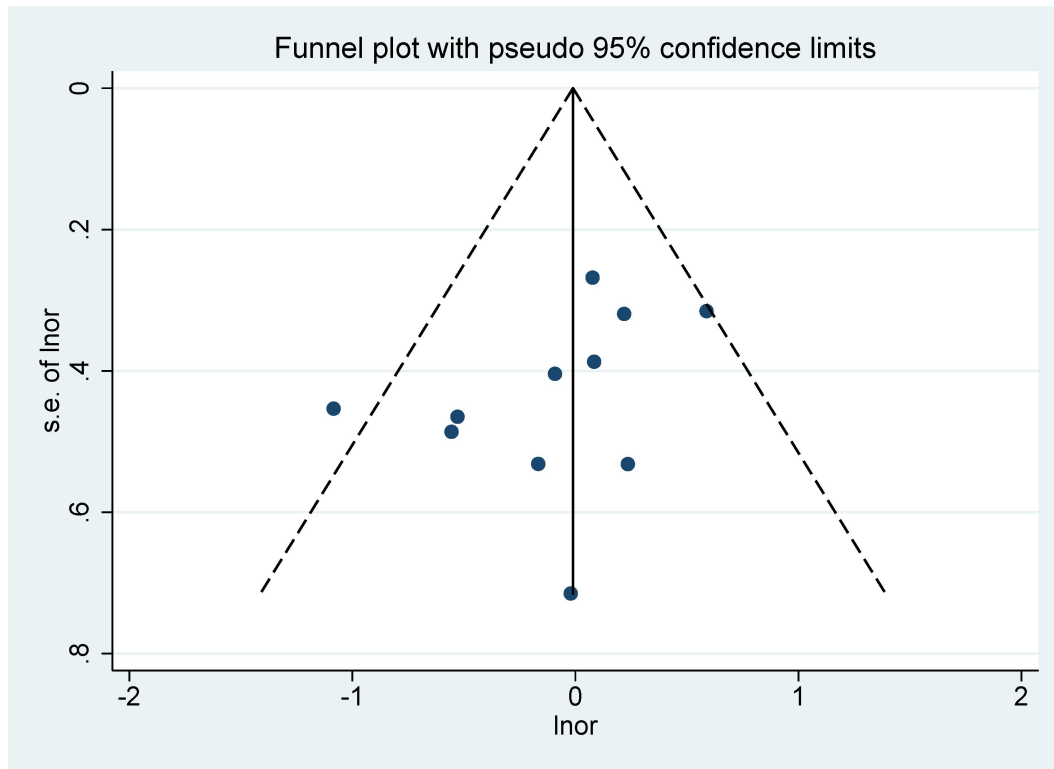

(b)

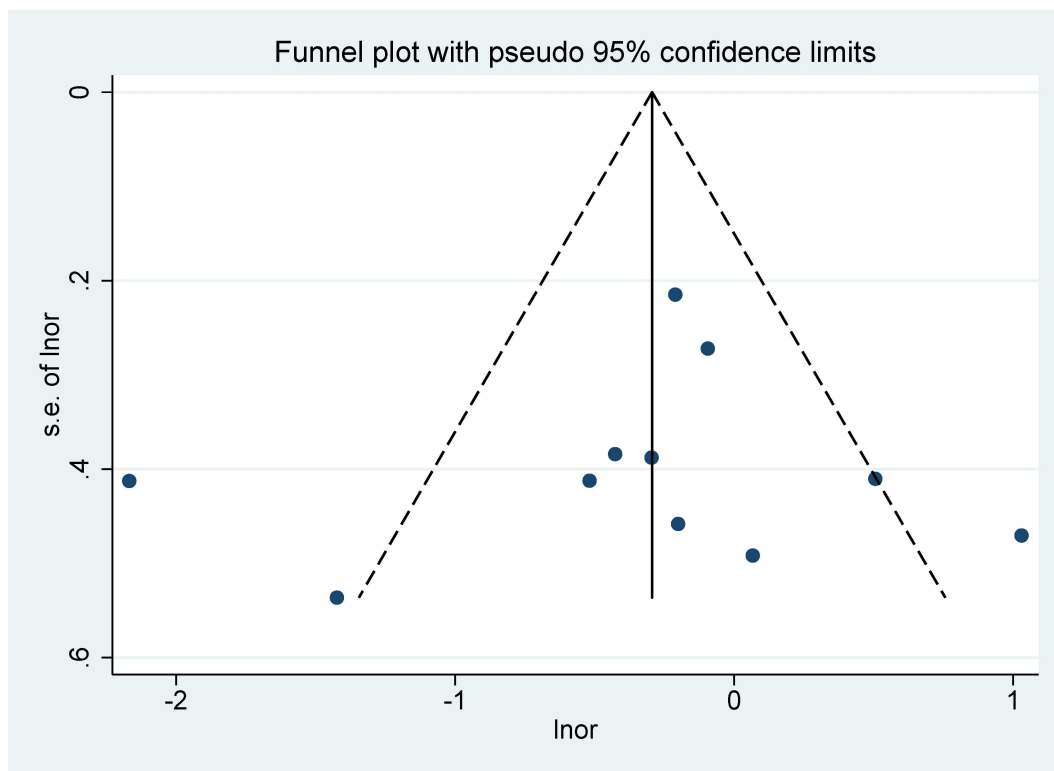

(c)

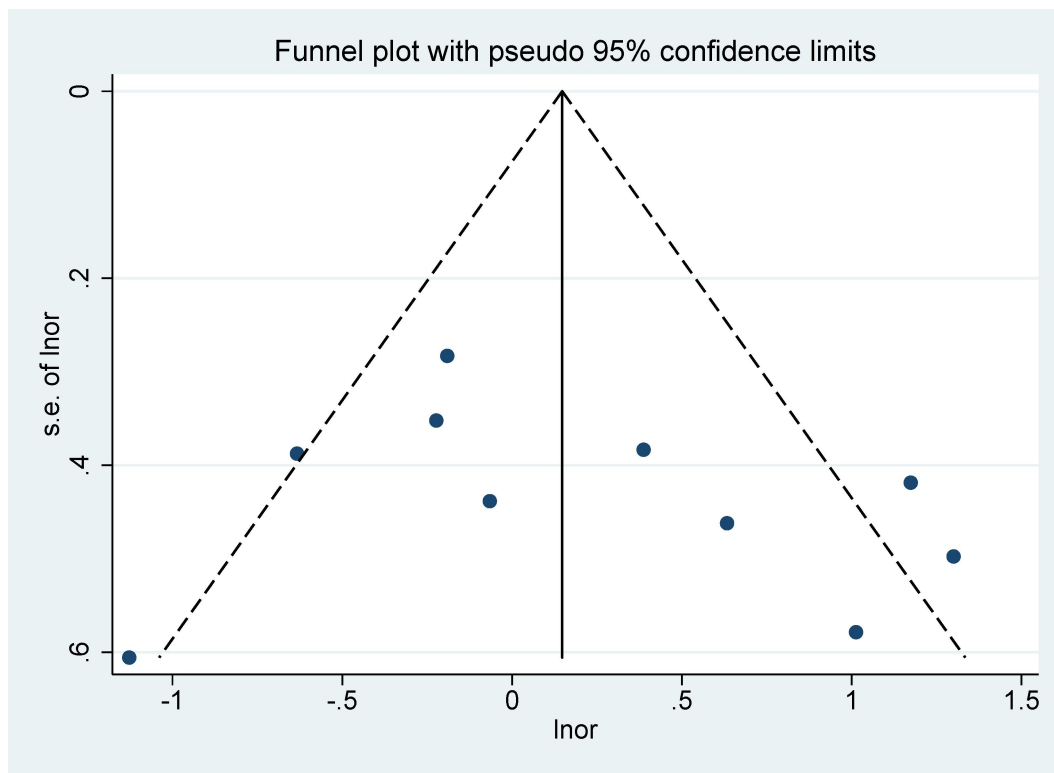

(d)

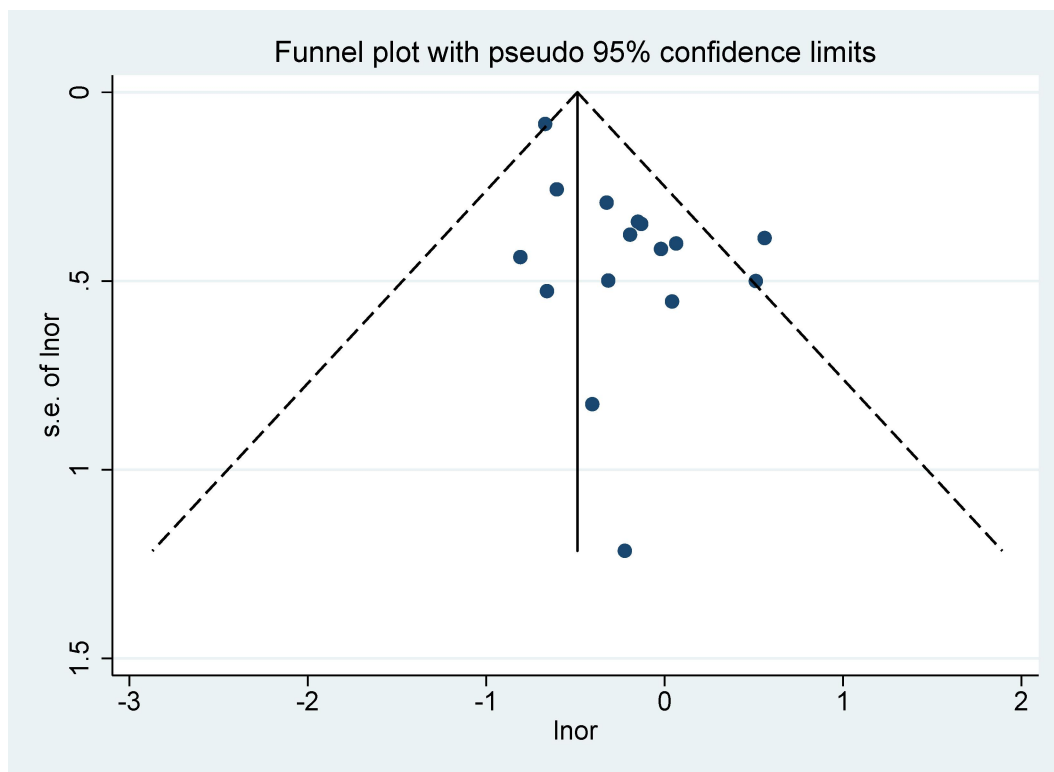

Supplement: Supplementary file 3 — Additional file 3. Funnel diagram. [file 12944_2023_1774_MOESM3_ESM.pdf]
