# Supplementary material for: Incidence and risk factors for cholelithiasis after bariatric surgery: a systematic review and meta-analysis
Source: Lipids Health Dis. 2023 Jan 14;22:5. doi: 10.1186/s12944-023-01774-7 (PMC9840335; doi:10.1186/s12944-023-01774-7)

**Additional file 5. publication bias test.** (a) DM; (b) Dyslipidemia; (c) Gender; (d) Hypertension; (e) Surgical procedure.

(a)

**
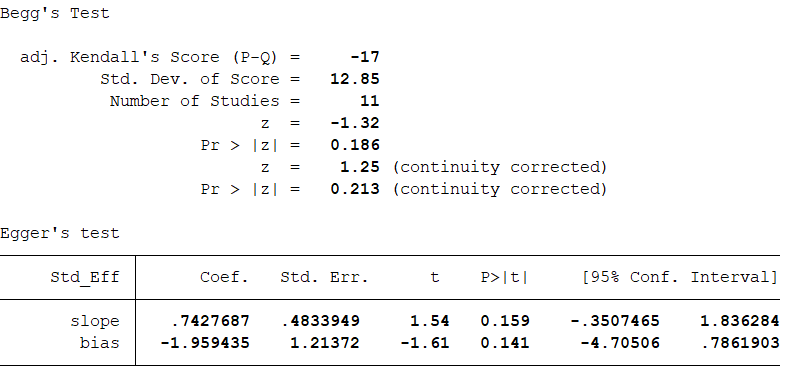
**

(b)

**
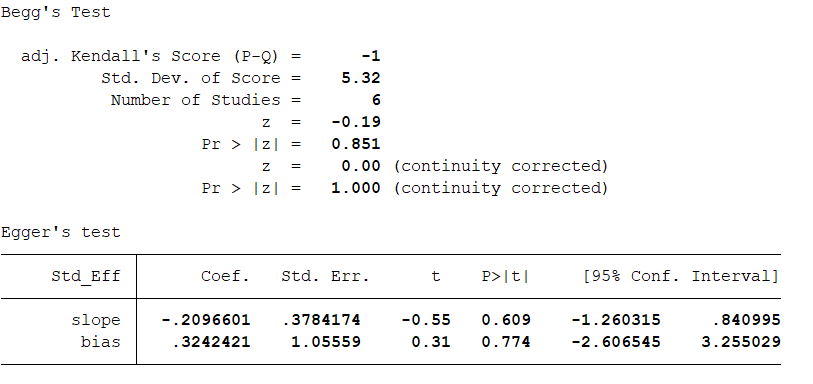
**

(c)

**
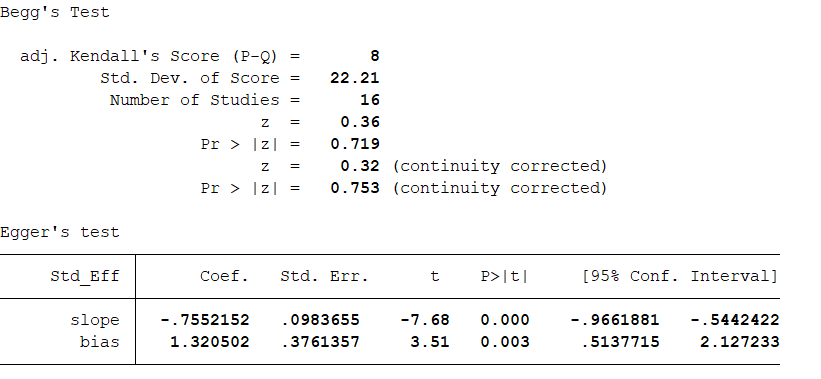
**

(d)

**
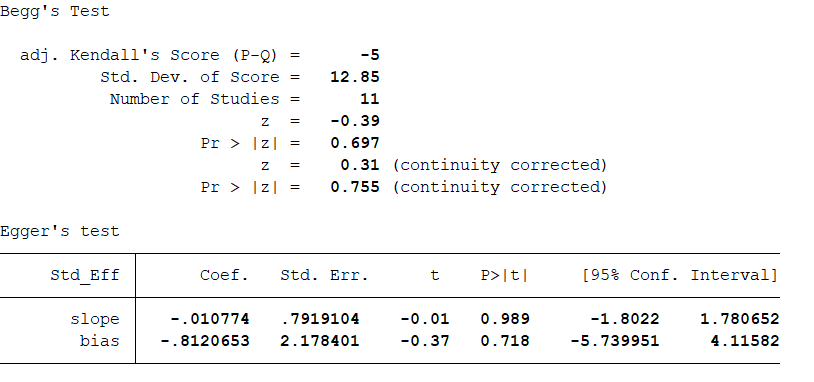
**

(e)


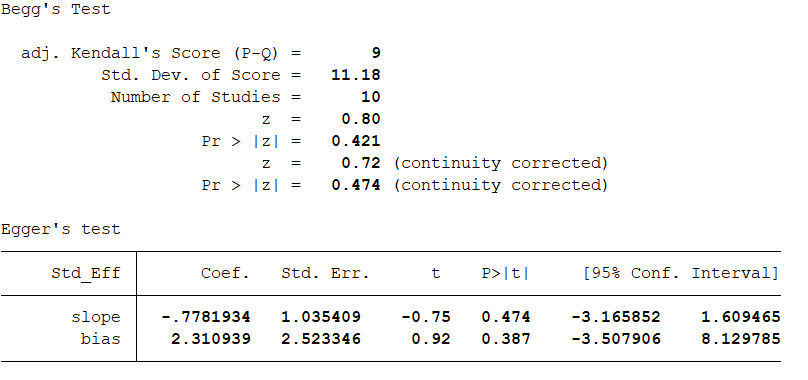

Supplement: Supplementary file 5 — Additional file 5. publication bias test. (a) DM; (b) Dyslipidemia; (c) Gender; (d) Hypertension; (e) Surgical procedure. [file 12944_2023_1774_MOESM5_ESM.docx]
